# Analysis of factors affecting organizational engagement between pre-hospital and hospital emergency departments: a qualitative study

**DOI:** 10.1186/s12873-025-01444-0

**Published:** 2025-12-16

**Authors:** Najmeh Baghian, Mohammad Sadegh Abolhasani, Somayeh Bagheri, Ali Zare Horoki, Adel Eftekhari

**Affiliations:** 1 Clinical Research Development Center, Shahid Rahnemoon Hospital, Shahid Sadoughi University of Medical Sciences, Yazd, Iran; 2 Trauma Research Center, Shahid Sadoughi University of Medical Sciences, Yazd, Iran; 3 Trauma Research Center, Shahid Sadoughi University of Medical Sciences, Yazd, Iran; 4 Department of Urology, School of Medicine, Shahid Sadoughi University of Medical Sciences, Yazd, Iran; 5 Department of Nursing, Meybod School of Medical Sciences, Shahid Sadoughi University of Medical Sciences, Yazd, Iran

**Keywords:** Emergency, Hospital, Pre-hospital, Organizational challenges, Organizational engagement, Health system

## Abstract

**Introduction:**

The provision of efficient and integrated emergency services constitutes a vital cornerstone for reducing mortality rates and improving clinical outcomes for patients and casualties within complex healthcare systems. Effective engagement between hospital-based and pre-hospital emergency services is crucial for the delivery of higher quality and more appropriate patient care. This study was conducted with the aim of analyzing the factors affecting organizational engagement between pre-hospital and hospital emergency departments.

**Methods:**

This was a qualitative study employing a conventional content analysis approach, conducted across pre-hospital emergency services and affiliated educational hospitals of Shahid Sadoughi University of Medical Sciences, Iran, in 2025. Participants included 38 experienced stakeholders in the field, encompassing managers, supervisors, physicians, nurses, and emergency medical technicians. These individuals were selected using purposive sampling until data saturation was achieved. Data were collected through semi-structured interviews and subsequently analyzed using MAXQDA software (Version 22). The credibility and trustworthiness of the findings were assessed according to Lincoln and Guba’s criteria.

**Results:**

Data analysis revealed that organizational interaction between pre-hospital and hospital emergency services faced significant barriers, primarily attributable to poor managerial coordination, cognitive and professional limitations, and gaps in clinical practice. Specifically, the lack of effective inter-organizational communication, weak leadership in conflict resolution, and insufficient structural and human resource support were identified as key managerial obstacles. In the domain of cognitive and professional limitations, negative attitudes, knowledge and skills gaps, and decision-making biases—often arising under conditions of high workload or limited information—were found to reduce mutual understanding and collaboration between personnel across the two sectors. Regarding clinical practice gaps, the absence of standardized patient handover protocols, inconsistencies in the implementation of care processes, and deficiencies in initial clinical assessments disrupted care continuity and diminished overall service quality.

**Conclusion:**

The findings of this study provide scientific guidance for decision-makers seeking to establish a more integrated, efficient, and safer emergency system. Enhancing organizational engagement within the emergency system necessitates a multifaceted and policy-driven approach, wherein policymakers play a pivotal role in reforming organizational structures, empowering human resources, and standardizing operational processes.

## Introduction

Today, the provision of comprehensive and high-quality healthcare services is widely regarded as a key indicator of societal development and progress [1]. Among these services, emergency care, as a fundamental pillar of the health system, plays a vital role in saving the lives of patients and casualties. These services comprise a series of immediate, specialized, and diagnostic interventions that, in many cases, determine survival and mitigate complications arising from illnesses and accidents [2]. The significance of timely and quality access to these services has led to emergency systems being recognized as the beating heart of healthcare delivery systems [3].

Emergency systems are structurally divided into two main components: pre-hospital and hospital-based, each playing a complementary and essential role in the chain of emergency service delivery. Pre-hospital emergency services, serving as the initial point of contact for patients with the healthcare system and providing life-saving care at the scene of an incident, are crucial for reducing mortality and disability through precise and timely interventions [3, 4]. By initiating essential treatment measures and ensuring the safe and rapid transfer of the patient to the hospital, pre-hospital services create the necessary readiness for hospital healthcare professionals. This readiness encompasses accurate and timely information regarding the patient’s condition, performed interventions, and potential needs, enabling the hospital to manage its resources and equipment more effectively [4]. Conversely, hospitals, equipped with advanced technology and specialized expertise, are responsible for patient stabilization, rehabilitation, and overall health improvement following initial care. The continuous improvement of emergency care quality remains a priority for every health system.

The interaction and coordination between pre-hospital and hospital emergency services, as two essential elements in the emergency care continuum, are of paramount importance. Despite their structural and functional differences, these two sectors strive to provide comprehensive, high-quality patient care through continuous and complementary interaction. However, research consistently demonstrates that challenges frequently impede their interaction and coordination, negatively impacting the quality and effectiveness of emergency services. Various studies indicate that effective coordination between hospital and pre-hospital emergency services can facilitate the continuous care process, increase patient satisfaction, reduce mortality, and lower overall healthcare system costs [4–6]. For instance, Eri et al. identified a lack of cooperation and interaction between pre-hospital emergency services and other organizations, including hospital emergency department staff, as a significant challenge faced by pre-hospital services [7]. Similarly, Jamshidi et al. identified individual capabilities, mutual understanding, and processes and infrastructure as the most critical components contributing to non-cooperation between hospital and pre-hospital emergency services in traffic accident scenarios [4]. Furthermore, the results of a study in Iran showed that insufficient coordination in terms of time and information exchange between the two emergency levels contributes to over 30% of treatment delays, which is associated with an increased likelihood of negative outcomes in trauma and cardiac patients [5].

Despite the complexity of managing the pre-hospital and hospital interface—a challenge underscored by high morbidity and mortality rates across the country—a significant empirical void exists concerning the specific organizational dynamics, barriers, and facilitators within the Iranian context. While existing national research has predominantly focused on isolated process metrics, such as response times (where studies indicate a non-trivial fraction of trauma victims succumb before definitive care), or general system performance indicators, there is a notable absence of in-depth, qualitative inquiry that systematically maps the nuanced inter-organizational engagement between Emergency Medical Services (EMS) and Emergency Department (ED) personnel. This deficit is critical because organizational friction, rather than purely clinical parameters, often dictates the throughput and quality of care transfer, directly impacting patient outcomes. Consequently, given the scarcity of evidence-based research that systematically analyzes the quality of organizational engagement between these two pivotal sectors in Iran—and recognizing the urgent need to translate findings into actionable policy to mitigate preventable losses—this study is both timely and necessary. It is noteworthy that the pre-hospital emergency services in Yazd city comprise 15 urban emergency stations under the coverage area of the university, which collectively transfer an average of over 25,000 patients annually to the three main teaching hospitals. This significant volume of patient transfer provides an ideal setting to observe the inherent tensions and coordination dynamics between the dispatch teams and the hospital receiving teams. The objective is the analysis of factors affecting organizational engagement between pre-hospital and Hospital emergency departments, aiming to construct a foundational framework for evidence-based interventions designed to enhance the overall effectiveness and continuity of the national emergency care system.

## Methods

### Study design and setting

This qualitative study employed a conventional content analysis approach in 2025, aiming to analyze the factors influencing organizational engagement between pre-hospital emergency services and the educational hospitals affiliated with Shahid Sadoughi University of Medical Sciences, Iran. The operational context involved the 15 urban emergency departments within the university catchment area, which collectively transport an average of more than 25,000 patients annually to the three main teaching hospitals. This substantial volume of patient transfers provides an ideal setting to observe the inherent tensions and coordination dynamics between hospital dispatch and admission teams.

The conventional content analysis methodology applied involved a systematic process structured across three distinct stages: open coding (initial data coding), organizing codes into subcategories, and finally, grouping subcategories into main categories to identify key patterns and overarching concepts within the textual data.

### Recruitment strategies and participants

Participants were selected using purposive sampling until the criterion of data saturation was met. This method is preferable in qualitative research as it allows the researcher to select individuals who possess the requisite knowledge, experience, and insight concerning the phenomenon under study (i.e., interdepartmental interactions of pre-hospital and hospital emergency departments). Data saturation was operationally defined as the point where new interviews ceased to yield entirely new concepts or insights. Selection criteria required participants to have sufficient knowledge and experience in managing and delivering services across both sectors. Participants were selected not solely based on their formal job position, but also on their direct practical experience. This was ensured by selecting individuals across strategic levels (managers), supervisory levels, and operational/clinical levels (physicians, nurses, technicians). The final sample comprised 38 experienced stakeholders distributed across three domains: Headquarters and Management (*n* = 11), Pre-hospital Emergency Services (*n* = 13), and Hospital Emergency Services (*n* = 14). This composition ensured a diversity of perspectives, ranging from operational staff up to high-level management. Furthermore, this sample size is considered acceptable in qualitative research aiming for an in-depth understanding of phenomena, particularly when determined by the criterion of content saturation.

### Data collection

The data collection process was meticulously designed to ensure the maximum richness and accuracy of the information obtained.

### Data collector and procedures

All stages of data collection—including establishing contact, conducting interviews, and recording information—were carried out by the main researchers of the study [8]. This approach guaranteed methodological consistency and allowed for direct familiarity with the specific research context.

1. **Pilot Phase**: Initially, three unstructured interviews were conducted with key informants. The purpose of these preliminary interviews was to identify commonly used terminology, understand the participants’ initial cognitive frameworks, and precisely determine the areas requiring deeper exploration.
2. **Main Interviews**: After refining the interview protocol based on the pilot phase findings, the main interviews were conducted in a semi-structured format.

- **Core Question**: The central focus of the interviews centered on the challenges, facilitators, and key barriers in organizational interaction between pre-hospital and hospital emergency services.
- **Guiding Questions**: These were flexible enough to allow for further probing into the unique experiences and detailed perspectives of each participant.

### Ensuring the accuracy and integrity of raw data

- **Interview Mode**: All interviews were conducted face-to-face to capture participants’ body language, tone, and the overall conversational context.
- **Recording and Transcription**: With participants’ formal consent, all sessions were audio-recorded and transcribed verbatim with high accuracy.
- **Interview Duration**: To ensure sufficient depth, each interview lasted between 60 and 90 min.

### Data analysis

Data analysis was conducted through a systematic approach grounded in the theoretical foundations of qualitative research.

### Analytical process

- **Analytical Method**: Conventional Content Analysis was employed, based on the theoretical and methodological framework proposed by Graneheim and Lundman. This approach is particularly suited to studies aiming to describe and interpret phenomena from participants’ perspectives.
- **Software**: To organize, code, and categorize the substantial volume of textual data, MAXQDA (version 22) was utilized.

### Analytical approach

- **Inductive Approach**: Data were analyzed entirely inductively, meaning that codes and categories were derived directly from the interview texts rather than imposed through any predetermined theoretical framework.
- **Inductive Steps**: Initially, the raw data were examined line by line to extract primary codes. These codes were then grouped into subcategories and ultimately abstracted into major categories (themes).

### Supporting findings with direct quotations

Instead of relying solely on abstract codes, findings were substantiated through direct quotations from participants to strengthen the validity and transparency of interpretations.

### Measures to ensure trustworthiness

To meet the methodological requirements of qualitative research, the four core criteria of Lincoln and Guba (1985) [9] were systematically addressed:

- **Credibility**: This signifies that the findings accurately represent the realities experienced by participants. This was ensured through In-depth Interviews (extended interviews of 60–90 min to ensure thorough extraction) and Member Checking (key participants reviewed summaries of findings or extracted codes to confirm the accuracy of interpretations).
- **Transferability**: This refers to the degree to which findings may be applied to similar contexts by external researchers. This was supported by providing a Thick Description, offering rich, detailed, and contextual descriptions of the study setting (universities, specific emergency units) and participants’ characteristics (roles, experience, managerial level) to enable readers to assess contextual comparability.
- **Dependability**: This concerns the stability and reproducibility of the research process. This was addressed through Comprehensive Documentation (meticulous documentation and time-stamping of all research stages) and Double Coding (Texts was coded independently by two researchers, and inter-coder reliability (Cohen’s Kappa = 0.82) was calculated to confirm consistency.
- **Confirmability**: This involves minimizing researcher bias and ensuring the objectivity of findings. This was achieved via Peer Debriefing (regular sessions with an external qualitative methodology expert to verify interpretations were grounded in data) and maintaining an Audit Trail (comprehensive, traceable records of all coding stages from raw codes to final categories to allow independent review).

## Results

Table 1 show cases a diverse and extensive composition of study participants (*n* = 38), encompassing three domains: Headquarters and Management (11 individuals), Pre-hospital Emergency Services (13 individuals), and Hospital Emergency Services (14 individuals).

**Table 1:** Characteristics of interview participants

Field of Activity	Position	Educational Background	Number
Headquarters and Management	Deputy of Treatment of the University, Head of the University’s Emergency Unit, Hospital President, Hospital Manager, Nursing Office Manager	Specialist Physician (4), General Practitioner (3), Ph.D. in Public Administration (1), Master of Nursing (3)	11
Pre-hospital Emergency	Head of Pre-hospital Emergency, Deputy of Technical and Operations of Emergency, Manager and Expert in Charge of the Provincial Emergency MCMC Center, Head of the Provincial EOC Unit, Emergency Dispatch Physician, Pre-hospital Emergency Supervisor, Dispatch Unit Manager, Public Education Unit Manager, Emergency Medical Technician	MPH in Crisis Management, Disasters and Emergencies and Fellowship in Health Networks Management (1), General Practitioner (3), Ph.D. in Management (1), Nursing Specialist and Senior Specialist (3), Emergency Medical Technician and Specialist (5)	13
Hospital Emergency	Head of Hospital Emergency, Emergency Medicine Physician, Hospital Emergency Supervisor/Head Nurse and Nurse, Triage Unit Nurse	General Practitioner (3), Emergency Medicine Specialist (4), Nursing Specialist (7)	14

The Table 2 provides a comprehensive and detailed summary of the findings from the qualitative data analysis concerning the organizational interaction challenges between EMS and ED. These findings are structured into three main categories of challenges: Management Challenges, Cognitive and Professional Constraints, and Gaps in Clinical Practice. The ordering of the main categories and subcategories within this table is based on the frequency of codes and priority ranking derived from the qualitative data, ensuring the most critical barriers are listed first.

Based on the qualitative analysis, weakness in inter-organizational communication (especially the failure to transfer vital information and the lack of standard channels) and attitudinal conflict between the two groups, along with weaknesses in clinical processes, were identified as the most recurrent and fundamental barriers.

**Table 2:** Factors affecting organizational interaction between prehospital emergency services and hospitals

Main Categories	Sub-categories	Representative Codes / Supporting Quotes
Management Challenges	Weaknesses in Inter-organizational Communication and Coordination	**Codes**: Absence of standard feedback and information flow; Difficulty contacting hospitals for admission coordination. **Quote**: “There’s no single channel—by the time we reach the ER, they often say ‘we were not informed.”
	Structural–Support Barriers	**Codes**: Shortage of space and triage facilities; Lack of ICT connectivity between systems. **Quote**: “Our HIS isn’t linked to 115, so patient information must be repeated several times.”
	Ineffective Management of Conflict	**Codes**: Weak leadership in problem resolution; Persistent inter-departmental tensions. **Quote**: “When disagreement occurs, no one mediates; the next shift inherits the same problem.”
	Ineffective Human Resource Management	**Codes**: Absence of rotation programs for emergency staff; Lack of transparent evaluation and career path. **Quote**: “Neither side fully understands the other’s workload—if we rotated once or twice a year, cooperation would be easier.”
Cognitive and Professional Constraints	Attitudinal Conflict	**Codes**: Prejudice and limited understanding of each other’s tasks and pressures; Negative stereotypes between hospital and EMS staff. **Quote**: “The hospital staff sometimes think we exaggerate the patients’ condition, while they don’t see what we face in the field.”
	Knowledge–Skill Gap	**Codes**: Insufficient familiarity with laws and transfer regulations; Lack of joint training to align skills and protocols. **Quote**: “We don’t have shared training—each side follows its own rules, so reports and expectations differ.”
	Decision-Making Bias	**Codes**: Relying on habit and proximity rather than clinical priority; Uneven patient distribution between hospitals. Quote: “Sometimes dispatch decides by distance, not by specialty—so a trauma patient may arrive where no trauma surgeon is on call.”
Gaps in Clinical Practice	Weakness in Clinical Processes	**Codes**: Lack of standard transfer and handover protocols; No joint mechanism for critical cases (e.g., CPR during transfer). **Quote**: “In emergencies like cardiac arrest, there’s no shared plan—the moment of handover gets chaotic.”
	Weakness in Clinical Performance	**Codes**: Inaccurate triage and initial assessment; Failure to follow patient handover steps thoroughly. **Quote**: “Sometimes patients wait with oxygen running, but vital signs aren’t re-checked before handover.”

### Managerial challenges

Managerial challenges pertain to critical issues in organization, leadership, and coordination between EMS and hospital emergency departments. The focus areas include communication deficiencies, ineffective conflict resolution, structural and support deficits, and inadequate human resource management, all of which collectively impede overall coordination and effectiveness. Identifying and systematically addressing these entrenched problems is a crucial prerequisite for enhancing mutual cooperation and elevating the quality of integrated emergency services.

### Weaknesses in inter-organizational communication and coordination

This subcategory represents the cornerstone of managerial dysfunction, as it directly governs the flow of critical information during patient transfer. Extracted codes clearly identify the most significant deficiency as the “failure to transmit vital patient data” and “difficulties in coordinating hospital acceptance of patients.” This problem originates from a fundamental lack of shared infrastructure, specifically the absence of a standardized, integrated communication system (such as a common Hospital Information System (HIS) or identical digital protocols) capable of reliably and automatically transferring records, preliminary findings, or the patient’s critical status to receiving hospital staff.

This communication breakdown is frequently exacerbated by the “absence of a constructive feedback loop.” Consequently, the pre-hospital team remains uncertain whether the information provided during handover was sufficient for the hospital team’s subsequent clinical decision-making. This flawed cycle, defined by the “lack of standard communication channels,” results in the redundant execution of information-gathering processes upon hospital arrival, thereby increasing clinical risk due to delayed access to accurate, historical data.

*…Our main problem is the lack of a shared*,* integrated communication infrastructure between departments*,* and this communication weakness challenges the patient transfer process because we lack common*,* inter-organizational standardized communication protocols… (P2*,*12)*

*…Our organizational culture is somewhat different from that of pre-hospital emergency services*,* and this discrepancy sometimes leads to misunderstandings in communication. It’s as if we don’t have a common language for coordination and collaboration*,* and ultimately*,* this affects staff behavior and even satisfaction… (P8*,*14*,*25)*

*…Hospital staff often believe we exaggerate patients’ conditions*,* while they don’t see the pressure we face on-scene. (P17)*

*…Sometimes*,* hospital staff receive incomplete data about the patient’s pre-hospital condition and must reconstruct the story from scratch. (P22*,* 26)*

### Structural and support barriers

These barriers are deeply rooted in the physical and technological resources required for true operational integration. Evidence highlights tangible physical shortcomings, including the “lack of space and facilities at the ambulance offloading area” and a shortage of critical resources such as “ICU/CCU beds,” which severely constrains hospital capacity. These limitations directly impose pressure on the pre-hospital team concerning patient distribution.

Beyond physical deficits, Information Technology (IT) barriers are prominent. One of the most significant obstacles is the “lack of connectivity between HIS and the pre-hospital emergency system.” This technological fragmentation not only reduces efficiency but also compromises optimal patient dispatch decisions due to incomplete or manually entered data, eventually leading to the “inappropriate allocation of beds based on relevant specializations.”

…Our main problem ranges from inadequate physical space to a shortage of ICU and CCU beds, and even a lack of ambulances. This scarcity of resources and facilities reduces our capacity to provide timely services and disrupts patient admission in hospitals….

*…It is truly unfair that resources*,* especially internal medicine beds and specialist physicians*,* are unevenly distributed across the province… (P17*,*20)*

*…The lack of connection between pre-hospital and hospital emergency systems has made coordination and vital information transfer problematic…(P 13*,*9)*

*…Even basic equipment*,* like stretchers or monitors*,* differ between services*,* so coordination during patient handover becomes chaotic. (P28)*

### Ineffective management of discord and conflict

When operational discrepancies between EMS and the Emergency Department (ED) escalate into tension, the lack of leadership to intervene and resolve these issues becomes a systemic organizational barrier. Findings explicitly point to a “lack of strong leadership to mediate disputes and foster inter-departmental relations.” This absence of proactive intervention allows dissenting opinions and workplace tensions to persist at the operational level, remaining latent and chronic rather than being formally addressed.

This mismanagement of conflict breeds negative dynamics in daily interactions, as staff feel their disagreements are neither acknowledged nor resolved through formal or trusted mechanisms. This can lead to decreased morale, unwillingness to collaborate on complex cases, and ultimately, the perpetuation of managerial impediments at lower policy levels.

*…Tension between our department and the hospital emergency department has always existed*,* and unfortunately*,* there’s no leadership to properly manage these differences. It’s as if everyone is playing their own tune*,* and this situation has prevented relations between departments from improving… (P10*,*15*,*33)*

*…To date*,* no joint meeting has been held with officials from both our and the hospitals’ emergency departments*,* with the aim of resolving or reducing tensions… (P2*,*3*,*21)*

*…There is no structured forum to reconcile misunderstandings between EMS and ED leaders. (P30)*

### Ineffective human resource management

These challenges center on skill development, occupational burnout, and employee reward structures. Findings reveal cross-cutting educational shortfalls, notably that “Emergency Department physicians and nurses have not completed pre-hospital emergency training,” which exacerbates the existing knowledge gap on both sides. This training deficit predisposes operational issues during patient handover.

From a retention standpoint, psychological burdens are significant, including “considerable psychological distress and stress in the workplace,” which compromises care quality. Furthermore, the organizational structure poorly supports high performance; existing codes refer to the “absence of a transparent performance evaluation system based on outcomes” that rewards inter-departmental cooperation, as well as the “lack of a defined career development and advancement path for personnel,” reducing the retention and motivation of key staff.

*…The occupational stress levels are exceptionally high*,* compounded by a perceived lack of managerial advocacy*,* which systematically erodes the sense of professional value… (P37*,* 32*,*14)*

*…There is a notable deficiency in objective staff performance evaluation*,* and compensation structures fail to differentiate merit-based rewards. Consequently*,* demonstrable professional contributions often remain unacknowledged… (P37*,* 12*,*14)*

*…The systemic absence of structured psychological support initiatives or mandated recovery policies fails to mitigate progressive staff burnout; personnel are driven to functional exhaustion without adequate systemic buffering. (P2*,* 10*,*24)*

*…Elevated attrition rates are the predictable outcome of sustained low staff motivation and the structural blockage of defined career progression pathways… (P8*,* 19*,*31)*

### Cognitive challenges

This category of challenges encompasses mental and processing barriers that affect individuals’ and groups’ ability to perceive correctly, analyze logically, and make effective decisions in the workplace. These barriers include attitudinal conflicts (differences in perspectives and values), knowledge-skill gaps (lack of necessary knowledge or skills for analysis), and decision-making biases (cognitive deviations in judgment), all of which influence how information is processed and the resulting outcomes.

### Attitudinal conflict

This subcategory indicates that many conflicts arise from fundamental differences in role perception and priorities, rather than mere skill deficits. Codes clearly point to “prejudice and insufficient understanding of each other’s roles and challenges.” This conflict surfaces when EMS personnel focus on rapid delivery and paramedic advanced on-scene care, while hospital personnel focus on managing constrained internal resources.

This attitudinal misalignment fuels the “presence of negative or stereotypical attitudes,” rooted in an “inadequate appreciation of the workload and limitations of each unit.” Specifically, reports indicate an “insufficient awareness of the significance and mandate of pre-hospital emergency services among academic authorities,” causing EMS perspectives to be undervalued at the strategic level, consequently affecting operational interactions.

*…Unfortunately*,* there is a lack of mutual understanding between hospital and pre-hospital emergency personnel. Both sides fail to grasp the workload and limitations of the other department*,* which leads to continuous misunderstandings and grievances… (P 12*,* 32*,* 15)*

*…Pre-hospital and hospital emergency services should be sequential*,* not adversarial. Sadly*,* this belief is absent*,* and pre-hospital emergency services are not recognized as the initial link for diagnosis and treatment… (P14*,*25*,*27)*

*…Pre-hospital teams are often judged as rushing or careless*,* but in reality*,* they work under unpredictable circumstances—we face constant resource uncertainty. (P22)*

*…Hospital physicians sometimes undervalue the EMS role*,* assuming we only transport patients without clinical reasoning. (P11)*

*…From our side*,* lack of trust in the accuracy of EMS reports sometimes causes tension*,* as detailed documentation is needed for subsequent medical decisions. (P18)*

### Knowledge–skill gap

This gap refers to a lack of shared prerequisite knowledge necessary for seamless service continuity, directly affecting the ability to establish an integrated workflow. A key finding is the “absence of a continuous joint training mechanism,” which prevents personnel in both sectors from becoming familiar with each other’s procedures and needs.

Specifically, this gap manifests in areas such as “EMS personnel’s lack of awareness regarding patient transfer regulations,” and conversely, hospital staff’s unfamiliarity with the advanced procedures performed prior to arrival. This shared knowledge deficit causes delays during the handover phase, as hospital staff must either repeat procedures already conducted in the ambulance or spend time reconciling documentation required by medical staff.

*…Our staff lack adequate information on the latest advancements and emergency protocols. Furthermore*,* we have insufficient awareness of the regulations and procedures related to patient transfer*,* and this mismatch in knowledge and skills sometimes results in non-compliance with legal job descriptions… (P11*,*16*,*19*,*24)*

*…There is a dire need for specialized and joint training to align everyone’s knowledge and skills. Some personnel lack the necessary professional qualifications*,* and this issue*,* coupled with a lack of awareness of regulations*,* creates serious challenges… (P19*,*12*,*13*,*25)*

*…We rarely have joint drills or training—each sector follows its own set of protocols. (P25)*

*…EMS personnel are insufficiently aware of the latest hospital triage or documentation rules*,* which leads to confusion at arrival. (P27)*

### Decision-making bias

This challenge focuses on subconscious cognitive errors frequently occurring under time constraints and environmental pressure. The most significant example cited is “decision-making based on experiential routines rather than clinical assessments.” This manifests as “prioritizing hospital proximity and speed of transport without considering appropriate specialization,” indicating the use of mental shortcuts instead of seeking the optimal treatment pathway.

These biases directly impact patient routing, resulting in a “suboptimal distribution of patients across city hospitals” in a non-specialized manner. This suggests that, even with the best intentions, the cognitive limitations of operational personnel can skew critical resource allocation decisions.

*…Dispatch decisions often rely on familiarity or proximity rather than specific specialty fit*. (P26)

*…At times*,* doctors request patients to be sent to their department without considering the system’s overall load.* [10]

*…Heavy workload causes some paramedics to rely on prior routines—even when patient conditions differ. (P19)*

*…Our hospital leaders should implement feedback mechanisms to analyze these transfer decisions collectively*,* not just leave them to individual experience. (P33)*

## Clinical challenges

### Weaknesses in clinical processes

This subcategory addresses the lack of mutually agreed-upon Standard Operating Procedures (SOPs) between the two sectors, which are vital for care continuity. Qualitative findings repeatedly emphasize the “lack of standardized patient handover and transfer protocols.” This includes issues such as “non-adherence to patient delivery procedures” and the absence of shared frameworks like SBAR or MIST that ensure structured information delivery.

Furthermore, codes indicate weaknesses in managing complex cases, including a “lack of a joint protocol for managing patients with special or critical conditions,” as well as a “lack of a shared process for equipment management during handover.” These procedural gaps cause patient care to depend heavily on individual knowledge rather than an integrated system.

*…Patient handovers are not performed correctly; for instance*,* a patient is not handed over to the triage unit*,* which indicates a weakness in the process… (P5*,*11*,*25*,*29)*

*…Especially in critical situations with many casualties*,* common protocols are needed to facilitate transfer and dispatch processes and guide us out of confusion… (P2*,*4*,*36)*

*…When ambulances bring in critical patients*,* shared protocols for equipment responsibilities and medical data transfer are lacking—this complicates accountability. (P37)*

### Weaknesses in clinical performance

This section addresses the practical execution and individual skills at the moment of patient delivery, which often result directly from training and procedural weaknesses. A key aspect is the “lack of diagnostic knowledge and initial patient assessment” by the dispatch team, which directly affects appropriate referral.

These end-stage weaknesses manifest as “incorrect application of pre-hospital and hospital triage.” Moreover, it is reported that due to the assumption that the hospital team “will re-do everything,” there is “neglect of the patient’s critical status during handover.” This suggests that personnel may become lax in fully executing their own assessments on-site, anticipating a redundant process at the hospital.

*…Sometimes*,* dispatch overestimates the patient’s stability*,* which delays necessary interventions after arrival. (P17*,*29)*

*…The absence of a thorough clinical examination upon a victim’s admission is indicative of weakness in our clinical performance*,* especially when the patient’s condition is truly critical… (P3*,*14*,*29*,*38)*

*…Clinicians assume that pre-hospital teams have already completed certain steps*,* so re-assessment is skipped*,* leading to blind spots in patient care. (P20)*

## Discussion

This qualitative inquiry systematically explored the organizational engagement challenges between EMS and ED. The analysis revealed that the barriers impeding seamless collaboration are concentrated within three interconnected, yet distinct, domains: Managerial Challenges, Cognitive and Professional Constraints, and Gaps in Clinical Practice.

The managerial domain encompasses structural and resource-related barriers, such as weaknesses in inter-organizational communication, inefficient conflict management, a lack of integrated information systems, and poor human resource development. These deficiencies fundamentally undermine coordination and accountability across the emergency chain and consequently compromise overall service quality. The cognitive and professional domain involves attitudinal and competency-related constraints arising from inconsistent understandings of professional roles, limited shared training opportunities, and decision-making biases that emerge under pressure. Attitudinal conflict between EMS and hospital staff, coupled with knowledge–skill gaps and the reliance on heuristic shortcuts in triage or patient allocation, actively hinders mutual trust, efficient teamwork, and collaborative decision-making. Finally, the clinical practice domain reflects operational deficiencies during the critical phases of patient handover and emergency management. Weaknesses in standardized clinical processes—such as absent or inconsistent transfer protocols—and deficiencies in personnel performance directly affect continuity of care, delay necessary treatment, and increase the risk of clinical error.

Collectively, these three domains form a dynamic system of interrelated barriers: managerial deficiencies invariably foster cognitive misunderstandings, which, in turn, lead directly to clinical inefficiencies. The subsequent sections provide a detailed discussion of each domain in relation to prior international and national research.

### Managerial challenges

This research investigated the organizational interaction challenges between pre-hospital emergency services and the affiliated educational hospitals of Shahid Sadoughi University of Medical Sciences in Iran. The study’s findings revealed that collaboration within the healthcare system is fraught with complex obstacles. These obstacles extend beyond mere managerial and structural issues to encompass attitudinal, knowledge-based, and clinical aspects. Analysis of interviews indicated that weaknesses in inter-organizational communication and the absence of a cohesive communication system are primary barriers to providing prompt and appropriate services to emergency patients. Furthermore, the lack of effective conflict management and dispute resolution mechanisms exacerbates tensions and reduces the level of cooperation between the two sectors. Previous studies have demonstrated that existing shortcomings in organizational interactions can have significant negative impacts on the quality of emergency services, increase mortality rates, and decrease patient satisfaction [4, 11]. These problems are particularly critical in situations where time is a vital factor in saving patients’ lives [12]. Sorani et al., in a qualitative study, identified issues related to management, human resources, and infrastructure as the most significant challenges, which aligns with the managerial and structural challenges in the present study [13]. Similarly, Jamshidi et al., in their examination of obstacles to collaboration between EMS and hospitals in patient handover following road traffic accidents, highlighted weaknesses in communication, differing viewpoints, and cultural barriers—findings consistent with the cognitive and clinical aspects of the current study [14]. In line with these findings, Hamidzadeh et al., in their study, identified organizational challenges such as human resource shortages, equipment scarcity, and structural-administrative issues as important factors influencing the interaction between pre-hospital emergency services and hospitals, which corresponds with the managerial challenges of the present research [15].

A key issue identified in this research is the inefficiency in inter-organizational communications, primarily stemming from the absence of integrated communication infrastructures and standardized protocols [16]. This problem leads to delays or errors in transmitting critical information, such as the patient’s clinical status, initial interventions performed, and treatment needs [17]. The lack of integration in Hospital Information Systems (HIS) between pre-hospital and hospital emergency departments hinders effective coordination and reduces the efficiency of the patient transfer process [18]. Global studies also confirm that the absence of shared information systems is a major obstacle to providing efficient emergency services [19–21].

Furthermore, organizational cultural differences between pre-hospital emergency services and hospitals, including misalignment in priorities and work methods, undermine collaboration and synergy [22]. These results are consistent with the findings of the present research, which indicate that a misaligned organizational culture can negatively impact staff satisfaction and the quality of services provided [23]. Additionally, the lack of strong and effective leadership in conflict management leads to intensified tensions and reduced cooperation among personnel [24]. Recent research suggests that effective leadership can significantly reduce conflicts by fostering empathy and improving communication [23, 25]. This is of great importance in emergency situations that require rapid and coordinated decision-making [26]. Bijani et al., in their study on the challenges faced by pre-hospital emergency personnel in mass casualty incidents, point to distinct organizational cultures, communication problems, bureaucratic structures, and resource competition as factors affecting organizational interaction between pre-hospital emergency services and hospitals, which corresponds with the managerial and cognitive challenges of the present study [23].

Among other fundamental challenges identified are structural impediments, such as the scarcity of ICU and CCU beds, physical space limitations, and inadequate IT infrastructure [27]. Findings from other studies also indicate the impact of resource and infrastructure shortages on the reduced efficiency of emergency services. Furthermore, financial issues, including the high costs associated with air evacuations, place additional strain on the emergency services system [28, 29]. In this context, a qualitative study on the challenges faced by pre-hospital emergency personnel in managing mass casualty incidents highlighted a lack of structural resources and support as a primary factor diminishing efficiency. It was suggested that investment in shared infrastructure, such as Integrated Coordination Centers (EOC), could alleviate these problems [21]. This approach not only enhances productivity but can also contribute to a more equitable distribution of resources across the province, which was identified as a key barrier in the present study as well.

### Cognitive and professional constraints

Naseri et al., in their research, attributed deficiencies in pre-hospital and hospital emergency care to conflicts arising from unclear personnel roles, insufficient competencies, and communication gaps. This underscores the need for standardized training and explicit definitions of emergency competencies to improve efficiency and collaboration [5]. Additionally, Strandås et al. (2024) identified weak communication between medical staff and delays resulting from clinical challenges as significant factors influencing organizational interaction in pre-hospital and hospital emergency centers [19].

The absence of joint training programs for pre-hospital and hospital emergency personnel has led to a lack of mutual understanding regarding each other’s challenges and responsibilities [28]. This issue exacerbates occupational stress, feelings of undervaluation, and decreased motivation among staff [29]. Recent studies suggest that implementing joint training programs and regular performance evaluations can mitigate these problems and foster team spirit [23, 26]. A knowledge-skill gap represents one of the primary challenges identified in this research. The unfamiliarity of some pre-hospital emergency technicians with patient transfer protocols leads to incorrect decision-making [30, 10]. Continuous training and the use of clinical simulations can help bridge this gap. Moreover, a lack of professional competence in hospital emergency departments, such as the improper execution of triage processes, negatively impacts the quality of care provided. Cognitive biases in decision-making, such as selecting an inappropriate hospital for patient transfer, are other obstacles that often occur under job pressures, traffic limitations, or a lack of up-to-date information on hospital capacities. The utilization of AI-based decision support systems can significantly reduce these errors. Additionally, multitasking and technician fatigue contribute to the escalation of incorrect decisions. A qualitative study on information barriers between EMS and hospital emergency departments identified 12 major barriers, including cognitive biases and a lack of joint training, suggesting the implementation of standardized protocols to reduce these biases [31]. These findings emphasize that integrating inter-organizational training not only enhances knowledge but can also help reduce decision-making biases, which is particularly important in the Iranian context, given resource limitations. Safik-Kikale et al., in their study, described factors influencing emergency technicians’ decision-making at the scene, including societal culture, organizational culture, authority dynamics, and the role of supervisors. These elements impact communication, decision-making, and the overall effectiveness of pre-hospital and hospital emergency services [32]. Similarly, Mantler and Hertzog identified factors influencing organizational interaction as including communication efficiency, data transfer reliability, usability of mobile computing systems, team training, and the integration of telemedicine programs [33].

### Gaps in clinical practice

From the perspective of clinical challenges, the findings of this study indicate that weaknesses in clinical processes, such as the absence of standardized protocols for managing critical patients, can lead to medical errors and delayed care. This issue aligns with international studies that emphasize how poor clinical coordination between pre-hospital and hospital settings can reduce survival rates [16]. To address this challenge, it is recommended that integrated national protocols be developed, including patient handover checklists and equipment management guidelines. Furthermore, weaknesses in clinical performance, such as inaccurate initial assessments, can be rectified through simulated training programs and regular performance evaluations, an approach validated in recent qualitative studies on patient safety in the pre-hospital environment [23]. Jamshidi et al., in their study, identified three main categories influencing collaboration: individual capabilities, the development of mutual understanding, and infrastructure and processes, all of which resonate with the cognitive and clinical challenges investigated in the present research [4]. Collectively, these challenges not only impact the quality of services but can also lead to increased healthcare system costs and a decline in public trust.

### Strengths and limitations

The strengths of the present study include its utilization of a qualitative approach and the conducting of in-depth interviews with a broad spectrum of stakeholders. This methodology facilitated a deeper understanding of the real-world experiences of both EMS and hospital staff. Moreover, this research appears to be among the few systematic qualitative studies conducted in Iran concerning EMS-ED interactions that concurrently examine diverse managerial, cognitive, and clinical dimensions. Conversely, the primary limitation of this study is its focus on a single province (Yazd). This restricts the generalizability of the findings to the entire country. Therefore, it is recommended that future research involve sampling across multiple provinces and at a national level to explore a wider variety of experiences and challenges. Additionally, the reliance on a qualitative methodology may introduce researcher bias; however, efforts have been made to mitigate this through techniques such as member checking and peer debriefing.

### Conclusion and recommendations

The findings of this study highlight the imperative for policymakers and health system managers to implement targeted interventions aimed at comprehensively revising the structures, processes, and prevailing attitudes within the emergency medical services system. From a policy perspective, this research underscores the prioritization of investment in integrated communication infrastructures, the development of comprehensive human resource empowerment programs emphasizing joint training and fostering a culture of collaboration, and the formulation and oversight of national protocols and standards for all aspects of emergency care. Furthermore, equitable and optimal resource allocation, including the provision of specialized equipment and personnel, is another critical area requiring attention.

Recommendations are directly mapped to the three primary challenge domains identified:

1. **Addressing Managerial Challenges**:

The most significant managerial obstacles identified are the lack of digital integration and inefficiency in conflict resolution. To overcome these, **Structural and IT Reforms** must be prioritized. This necessitates the implementation of a unified, shared Hospital Information System (HIS) interface that is accessible by EMS for real-time viewing of bed availability and patient transfer logging. Furthermore, to manage persistent tensions, a formal, tiered conflict resolution committee involving mandatory participation from both sectors must be established to resolve disputes in a structured manner.

2. **Addressing Cognitive and Professional Constraints**:

To counter attitudinal conflicts, biases, and knowledge gaps among personnel, the focus must shift to **Culture and Competency Building**. This should be achieved through continuous, joint simulation-based training (especially for high-acuity trauma management) that brings EMS and hospital teams together. Developing standardized protocols for pre-briefing and post-debriefing between teams will also help align professional expectations and mitigate unconscious biases.

3. **Addressing Gaps in Clinical Practice**:

Clinical process deficiencies stem from the absence of unified transfer protocols and inconsistent documentation standards. The key solution here is **Clinical Standardization**. A national/regional standard protocol for patient handover (e.g., utilizing established frameworks like SBAR or MIST) must be developed and made mandatory, requiring sign-off from both sending and receiving personnel upon completion. Moreover, to ensure the sustainability of these standards, quarterly audits must be conducted to monitor adherence to these protocols.

By implementing these integrated recommendations, policymakers can move beyond merely documenting problems to actively engineering a resilient and cohesive organizational engagement within the emergency medical system.

Also; future research can evaluate the effectiveness of the proposed solutions (especially integrated information systems and joint training protocols) on objective indicators such as response time and survival rates by employing quantitative or interventional models.

## Data Availability

All analyzed data during this study are included in this Manuscript.
